# Natural products from food sources can alter the spread of antimicrobial resistance plasmids in Enterobacterales

**DOI:** 10.1099/mic.0.001496

**Published:** 2024-08-27

**Authors:** Ilyas Alav, Parisa Pordelkhaki, Judith Rodriguez-Navarro, Onalenna Neo, Celia Kessler, Ruth Jesujobalayemi Awodipe, Poppy Cliffe, Nivethanaa Pulavan, Huba L. Marton, Simon Gibbons, Michelle M. C. Buckner

**Affiliations:** 1 Institute of Microbiology and Infection, College of Medical and Dental Sciences, University of Birmingham, Birmingham, UK; 2 Department of Microbiology, Hospital de la Santa Creu i Sant Pau, Institut d’Investigació Biomèdica Sant Pau (IIB Sant Pau), Sant Quintıí 89, E-08041 Barcelona, Spain; 3 School of Dentistry, College of Medical and Dental Sciences, University of Birmingham, Birmingham, UK; 4 Warwick Medical School, University of Warwick, Coventry, UK; 5 Natural and Medical Sciences Research Center, University of Nizwa, P.O. Box 33, Birkat Al Mauz, Nizwa 616, Sultanate of Oman

**Keywords:** anti-plasmid, antibiotic resistance, conjugation, *E. coli*, *K. pneumoniae*

## Abstract

Antimicrobial resistance (AMR) poses a significant threat to global public health. Notably, resistance to carbapenem and extended-spectrum β-lactam antibiotics in Gram-negative bacteria is a major impediment to treating infections. Genes responsible for antibiotic resistance are frequently carried on plasmids, which can transfer between bacteria. Therefore, exploring strategies to prevent this transfer and the prevalence of AMR plasmids is timely and pertinent. Here, we show that certain natural product extracts and associated pure compounds can reduce the conjugation of AMR plasmids into new bacterial hosts. Using our established high-throughput fluorescence-based flow cytometry assay, we found that the natural products were more active in reducing transmission of the IncK extended-spectrum β-lactamase-encoding plasmid pCT in *Escherichia coli* EC958c, compared to *Klebsiella pneumoniae* Ecl8 carrying the IncFII carbapenemase-encoding plasmid pKpQIL. The exception was the natural product rottlerin, also active in *K. pneumoniae*. In classical conjugation assays, rottlerin also reduced the conjugation frequency of the IncFII *bla*_NDM-1_ carrying plasmid pCPE16_3 from a clinical *K. pneumoniae* isolate. Our data indicate that the natural products tested here, in their current molecular structure, reduced conjugation by a small amount, which is unlikely to achieve a large-scale reduction in AMR in bacterial populations. However, certain natural products like rottlerin could provide a foundation for further research into compounds with effective anti-plasmid activity.

## Introduction

Antimicrobial resistance (AMR) is a growing global problem, with resistant bacteria causing increasing numbers of difficult-to-treat infections, leading to increased morbidity and mortality. Recently, the World Health Organization has designated third-generation cephalosporin-resistant and carbapenem-resistant Enterobacterales as a critical priority for the development of novel therapies [1]. Mobile genetic elements, such as plasmids, have resulted in the widespread dissemination of extended-spectrum β-lactams (ESBLs) and carbapenemases amongst these organisms. Plasmids are self-replicating pieces of DNA that can carry a variety of accessory genes, including multiple AMR and/or virulence genes [2,3]. Conjugative plasmids encode all the necessary machinery to mediate their transmission from one bacterial cell to another through a process called conjugation [4]. Conjugative transfer of a plasmid into a new host has the potential to turn a drug-susceptible strain into a multidrug-resistant strain and, in worst-case scenarios, also a hypervirulent strain [5]. Therefore, research into these mobile genetic elements is of considerable importance.

The most common plasmid type isolated from animal and human sources is IncF plasmids [2]. These plasmids commonly carry multiple genes encoding AMR determinants, such as ESBLs and carbapenemases [6,9]. One example is the 114 kb IncFII plasmid pKpQIL, which carries the *bla*_KPC-3_ carbapenemase, *bla*_TEM-1_ β-lactamase, and heavy metal resistance genes [10]. The KPC-3 carbapenemase confers resistance to penicillin, cephalosporin, and carbapenem antibiotics, and is resistant to standard β-lactamase inhibitors, such as clavulanic acid, tazobactam, and sulbactam [11,13]. pKpQIL was first identified in an extensively drug-resistant epidemic strain of *Klebsiella pneumoniae* isolated in Israel between 2006 and 2008 [10,14]. Since then, pKpQIL and its variants have been reported worldwide in various species of Enterobacterales [15,16]. In addition to IncF plasmids, another important vector of ESBLs in *Escherichia coli* is IncK plasmids like pCT, which carries the *bla*_CTX-M-14_ ESBL gene [17]. The CTX-M-14 ESBL confers resistance to several clinically important third-generation cephalosporins, such as cefotaxime, ceftriaxone, and cefpodoxime [18]. pCT-like plasmids have been identified in human and animal *E. coli* isolates from Australia, Asia, and Europe [17]. Furthermore, IncK plasmids have contributed to the dissemination of the *bla*_CTX-M-14_ ESBL gene in the UK and Spain [19,21].

Some approaches have focused on different methods to remove plasmids from bacterial hosts (curing agents), and others have focused on preventing plasmid transfer (conjugation inhibitors); broadly speaking, such anti-plasmid approaches are gaining interest [22,23]. The search for and use of anti-plasmid compounds capable of curing plasmids or inhibiting conjugative plasmid transfer are ongoing [24,27]. Natural products have historically played a significant role in drug discovery owing to the extensive structural diversity and complexity of chemical compounds [28]. Certain food products and their bioactive constituents possess diverse physiological effects. For example, ginger has been reported to have protective effects on gastrointestinal, nervous, and cardiovascular systems [29], displays antimicrobial effects [30], and has been associated with improved outcomes in fatty liver diseases [31]. Similarly, black pepper and turmeric extracts have been reported to have diverse physiological effects *in vitro* and *in vivo*, including anti-tumorigenic, anti-diarrhoeal, antioxidant, and antimicrobial effects [32,33]. The kamala tree (*Mallotus philippensis*) and its fruit have been traditionally used to treat parasitic infections and are reported to have antimicrobial, antioxidant, and anti-inflammatory properties [34]. Therefore, the wealth of diverse phytochemicals in natural products could offer compounds with anti-plasmid activity.

Here, we performed a screen for natural products with anti-plasmid activity (with either plasmid curing or conjugation inhibitor activity) by measuring the effects of bioactive plant extracts and bioactive compounds from black pepper, ginger, cashew nuts, and kamala on plasmid conjugation in *E. coli* and *K. pneumoniae*.

## Methods

### Natural product extracts and compounds

Extracts were produced by extracting 10 g of powdered plant material with chloroform (200 ml) overnight at room temperature. Extracts were then concentrated under vacuum and stored in a freezer at −20 °C until use. The compounds 6-gingerol, capsaicin, anacardic acid, and rottlerin were purchased from Merck, UK. Extracts or compounds were dissolved in DMSO, and DMSO vehicle controls were used at the same volume throughout.

### Bacterial strains

The bacterial strains used are described in Table 1. Unless stated otherwise, all strains were grown in Luria–Bertani (LB) broth supplemented with the appropriate antibiotics and incubated at 37 °C with aeration.

**Table 1.** Bacterial strains and antibiotics used in this study

Strain no.	Strain description	Antibiotic selection	Reference
EC18	*E. coli* EC958c belonging to the ST131 clonal group	100 µg ml^−1^ rifampicin	[24]
EC24	EC18 carrying IncK plasmid pCT*gfp* (*gfp-aph* inserted into the *bla*_CTXM-14_ gene)	50 µg ml^−1^ kanamycin	[24]
EC25	EC18 with chromosomal *mCherry* (*mCherry-aph* cassette inserted between *putPA* on the chromosome)	50 µg ml^−1^ kanamycin	[24]
KP10	Carbapenem-resistant *K. pneumoniae* clinical isolate belonging to the ST14 clonal group carrying the IncF pCPE16_3 plasmid	2 µg ml^−1^ doripenem	[36]
KP17	Wild-type *K. pneumoniae* Ecl8 (derivative of human clinical isolate)	100 µg ml^−1^ rifampicin	[24]
KP18	KP17 with chromosomal *mCherry* (*mCherry* cassette inserted between *putPA* on the chromosome)	50 µg ml^−1^ kanamycin	[24]
KP19	KP17 carrying IncF plasmid pKpQIL*gfp* (*gfp-aph* cassette inserted into the *bla*_KPC-3_ gene on pKpQIL)	50 µg ml^−1^ kanamycin	[24]
KP20	*K. pneumoniae* ATCC 43816R containing *hph* inserted into the chromosomal *bla*_SHV-1_	300 µg ml^−1^ hygromycin	[36]

### High-throughput screening of extracts and compounds

The transmission of pCT*gfp* in *E. coli* EC958c and pKpQIL*gfp* in *K. pneumoniae* Ecl8 in the presence of natural product extracts and compounds was measured by flow cytometry as previously described [24]. Briefly, 1 ml of the overnight cultures of the donor (*E. coli* EC958c with pCT*gfp* or *K. pneumoniae* Ecl8 with pKpQIL*gfp*) and the recipient (*E. coli* EC958c or *K. pneumoniae* Ecl8 with chromosomal *mCherry*) strains were pelleted, washed in sterile PBS, and diluted to an OD_600_ of 0.5. Equal volumes of donor and recipient strains were mixed to give a donor-to-recipient ratio of 1 : 1. A 20 µl volume of the donor–recipient mix was inoculated into 180 µl of LB broth supplemented with a final concentration of natural product extract or compound in a 96-well round bottom plate (Corning, USA). The same volume of DMSO was also added to 180 µl of LB broth as vehicle control. The plate was incubated at 37 °C with gentle agitation (∼100 r.p.m.) for 24 h (*E. coli)* or 6 h (*K. pneumoniae)*. Following incubation, 20 µl was removed and serially diluted 1 : 1000 in filter-sterilised Dulbecco’s PBS (Merck, UK). Samples were analysed on the Attune NxT acoustic focusing flow cytometer with Autosampler (Thermo Scientific, USA). GFP emission was collected using the BL1-H channel and the mCherry emission was collected using the YL2-H channel. For each sample, 10 000 bacterial events were recorded. Plasmid conjugation was measured by quantifying the number of green fluorescent protein (GFP)-positive bacteria (donor), mCherry-positive bacteria (recipient), and GFP-positive/mCherry-positive bacteria (transconjugants). Gating strategies were exactly as previously described [24]. The conjugation frequency was calculated as the number of dual fluorescent bacterial events (transconjugants) divided by the number of mCherry-positive-only events (recipients). Three independent experiments were carried out, each one consisting of four biological replicates.

### Antimicrobial susceptibility testing

The antimicrobial susceptibility of bacterial strains to natural product compounds was determined using the broth microdilution method [35]. Briefly, overnight cultures grown in LB broth (~1×10^9^ c.f.u. ml^−1^) were diluted to 1×10^6^ c.f.u. ml^−1^ in LB broth. A 10 mg ml^−1^ stock solution of 6-gingerol, anacardic acid, capsaicin, and rottlerin was prepared fresh on the day of each experiment using DMSO as a diluent. A 1024 µg ml^−1^ working stock solution of each compound was prepared in LB broth. In a round bottom 96-well plate, 100 µl of the working stock solution was inoculated into the first column and 50 µl of LB broth was dispensed into the rest of the wells. A 50 µl volume was removed from the first well and added to the second and mixed, and this process was repeated across the columns to give a concentration range from 1024 down to 1 µg ml^−1^. For each strain, a 50 µl volume of the 1×10^6^ c.f.u. ml^−1^ was dispensed into wells of a single row. This resulted in a final concentration range of 512 down to 0.5 µg ml^−1^. The plates were incubated for 18 h at 37 °C. The minimum inhibitory concentration was determined as the lowest concentration of a compound that visibly reduced the growth of bacteria.

### Growth kinetic assays

Overnight cultures of bacteria grown in LB broth (~ 1×10^9^ c.f.u. ml^−1^) were diluted to a starting inoculum of 1×10^6^ c.f.u. ml^−1^ in a 96-well flat bottom plate (Corning, USA). Where appropriate, the test strains were diluted in LB broth supplemented with pure compounds. Concentrations of the compounds tested were 256 µg ml^−1^ 6-gingerol, 256 µg ml^−1^ anacardic acid, 128 µg ml^−1^ capsaicin, and 128 µg ml^−1^ rottlerin. The same volume of DMSO was used as a vehicle control to ensure that DMSO did not adversely affect bacterial growth. The optical density at 600 nm (OD_600_) was measured every 30 min for 24 h at 37 °C with shaking (200 r.p.m.) using the FLUOstar OMEGA plate reader (BMG Labtech, Germany). Three independent experiments were carried out, each consisting of three biological replicates.

### Liquid broth conjugation with clinical isolate

The *K. pneumoniae* clinical isolate carrying the IncF plasmid pCPE16_3*bla*_NDM-1_ (KP10) was paired with the hygromycin-resistant *K. pneumoniae* ATCC 43816R recipient strain (KP20) in liquid broth as previously described [36]. Briefly, KP10 and KP20 cultures were grown overnight, and sub-cultures were prepared in 5 ml LB broth (1% inoculum) and grown to an OD_600_ of ∼0.5. Then, 1 ml of cultures were pelleted, and media were replaced with LB broth to adjust the OD_600_ to 0.5. The donor (KP10) and the recipient (KP20) were mixed at a 1 : 10 ratio alongside control single strains. The donor, recipient, and mixed cultures were separately diluted 1 : 5 in LB broth containing a final concentration of 100 µg ml^−1^ of the natural compound or the same volume of DMSO as vehicle control and these were incubated statically at 37 °C for 1 h. Corresponding dilutions were plated onto LB agar to assess cell viability and selective media to determine donor-to-recipient ratios and transconjugant production. Plates were incubated at 37 °C overnight. Transconjugant colonies carrying pCPE16_3*bla*_NDM-1_ were selected on LB agar supplemented with 300 µg ml^−1^ hygromycin B (PhytoTech Labs, USA) and 2 µg ml^−1^ doripenem (Merck, Germany). Conjugation frequencies were calculated as the number of transconjugants per recipient. Data shown are the mean±standard deviation of three independent experiments, each carried out with four biological replicates.

### Statistical analysis

**Fig. 1.**
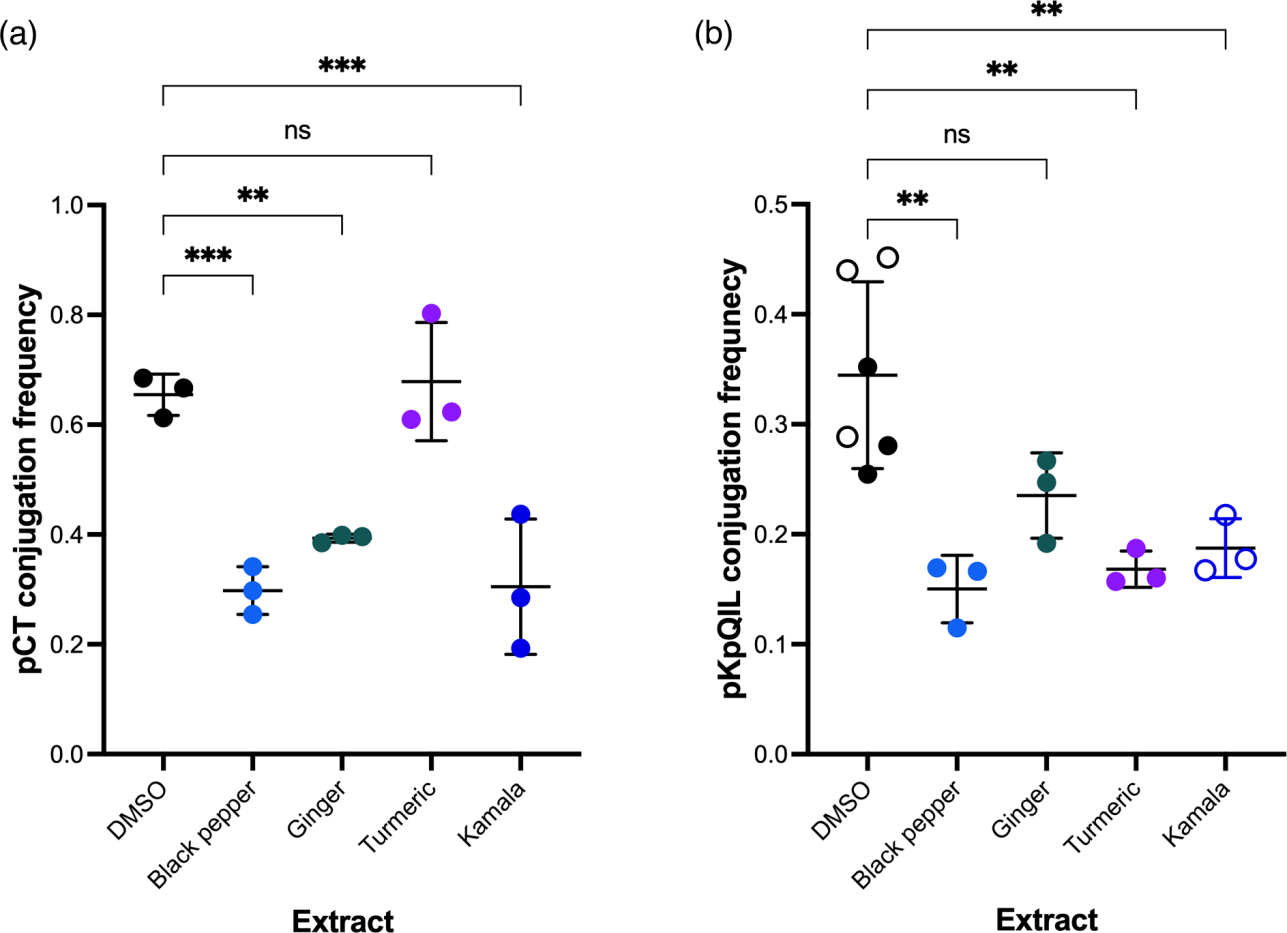
The effect of natural product extracts on plasmid conjugation. (**a**) Conjugation frequency of pCT*gfp* from EC24 (*E. coli* EC958c carrying pCT*gfp*) to the recipient EC25 (*E. coli* EC958c mCherry). (**b**) Conjugation frequency of pKpQIL*gfp* from KP19 (*K. pneumoniae* Ecl8 carrying pKpQIL*gfp*) to the recipient KP18 (*K. pneumoniae* Ecl8 mCherry). The donor and recipient strains were mixed at a 1 : 1 ratio and then added to LB broth supplemented with 0.25 mg ml^−1^ of extract or an equal volume of DMSO as vehicle control. Conjugation assays were carried out for 24 h (*E. coli*) and 6 h (*K. pneumoniae*) at 37 °C. For *K. pneumoniae*, the kamala extract was tested in separate experiments and, therefore, was compared to its own DMSO control (indicated by clear circles). The conjugation frequencies were calculated as the number of dual fluorescent bacteria (transconjugants) divided by the mCherry-positive-only bacteria (recipients). Data presented are mean±standard deviation of three independent experiments, each consisting of four biological replicates. The conjugation frequencies of the plasmids treated with DMSO were compared to those treated with the extracts using one-way ANOVA with Dunnett’s test to correct for multiple comparisons. Significantly different results are indicated with ** (*P*≤0.005) or *** (*P*≤0.001). ns, not significant.

In Figs1^3^, the mean of each treatment group was compared to the mean of the DMSO control using one-way ANOVA followed by Dunnett’s test to correct for multiple comparisons. In Fig. 4, the mean of the DMSO control was compared to the mean of the treatment group using two-tailed unpaired *t*-tests. All statistical analyses were performed using GraphPad Prism version 10 for MacOS (GraphPad, San Diego, CA USA)http://www.graphpad.com. Only *P*-values ≤0.05 were considered statistically significant.

## Results

### Extracts from natural products can reduce plasmid conjugation

Given the highly bioactive properties of certain food products, it was decided to test the activity of extracts from black pepper, ginger, turmeric, and kamala using a previously developed [24] flow cytometry assay for monitoring the conjugation of pCT and pKpQIL in *E. coli* EC958c and *K. pneumoniae* Ecl8, respectively (Fig. 1). In this assay, the donor and recipient cells can be differentiated using flow cytometry based on their GFP and mCherry fluorescence, respectively. As a result, transconjugant cells can be identified based on their dual fluorescence of GFP and mCherry. For *E. coli*, the conjugation assays consisted of a 1 : 1 mix of the donor EC24 (*E. coli* EC958c pCT*gfp*) and the recipient EC25 (*E. coli* EC958c mCherry) (Table 1), followed by a 24 h incubation at 37 °C. All extracts and pure compounds used in this study were dissolved in DMSO, therefore, their effects on plasmid conjugation were compared to treatment with the same volume of DMSO as vehicle controls. The conjugation frequency of pCT in *E. coli* was significantly reduced in the presence of black pepper (*P*<0.001), ginger (*P*=0.007), and kamala (*P*<0.001) extracts compared to the DMSO vehicle control (Fig. 1a). Turmeric extract did not have a significant impact (*P*=0.986) on pCT conjugation in *E. coli* (Fig. 1a).

For *K. pneumoniae*, the conjugation assays consisted of a 1 : 1 ratio of the recipient KP18 (*K. pneumoniae* Ecl8 mCherry) and the donor KP19 (*K. pneumoniae* Ecl8 pKpQIL*gfp*) (Table 1), followed by a 6 h incubation at 37 °C. In *K. pneumoniae*, the conjugation frequency of pKpQIL was significantly reduced in the presence of black pepper (*P*=0.001), turmeric (*P*=0.003), and kamala extracts (*P*=0.007) compared to the DMSO vehicle control. However, ginger extract had no significant impact (*P*=0.063) on pKpQIL conjugation in *K. pneumoniae* (Fig. 1b). Comparing the effects of the natural product extracts on pCT and pKpQIL conjugation frequencies, black pepper, and kamala extracts were effective against both plasmids, whilst ginger and turmeric had a plasmid-specific effect.

As the flow cytometry assay relies on the fluorescent markers to identify donor, recipient, and transconjugant cells, the impact of the extracts on GFP and mCherry fluorescence was also determined. All tested extracts significantly increased the number of non-fluorescent EC24 and EC25 cells after incubation compared to the DMSO control (Fig. S1a and Table S1, available in the online version of this article). This suggested that the decrease in the conjugation frequency of pCT in *E. coli* by the extracts could also be due to fluorescence interference. For KP18 and KP19, the black pepper, ginger, and kamala extracts had no significant effect on the number of fluorescent cells, while the turmeric extract significantly increased the number of non-fluorescent cells (Fig. S1b). Turmeric extract reduced the number of fluorescent KP18 and KP19 cells, suggesting the presence of compounds that could be interfering with the fluorescence of GFP and mCherry. However, it should be noted that the conjugation frequency is calculated as the proportion of dual fluorescent cells per fluorescent recipient cell.

### Pure compounds from natural product extracts have a moderate effect on plasmid conjugation

**Fig. 2.**
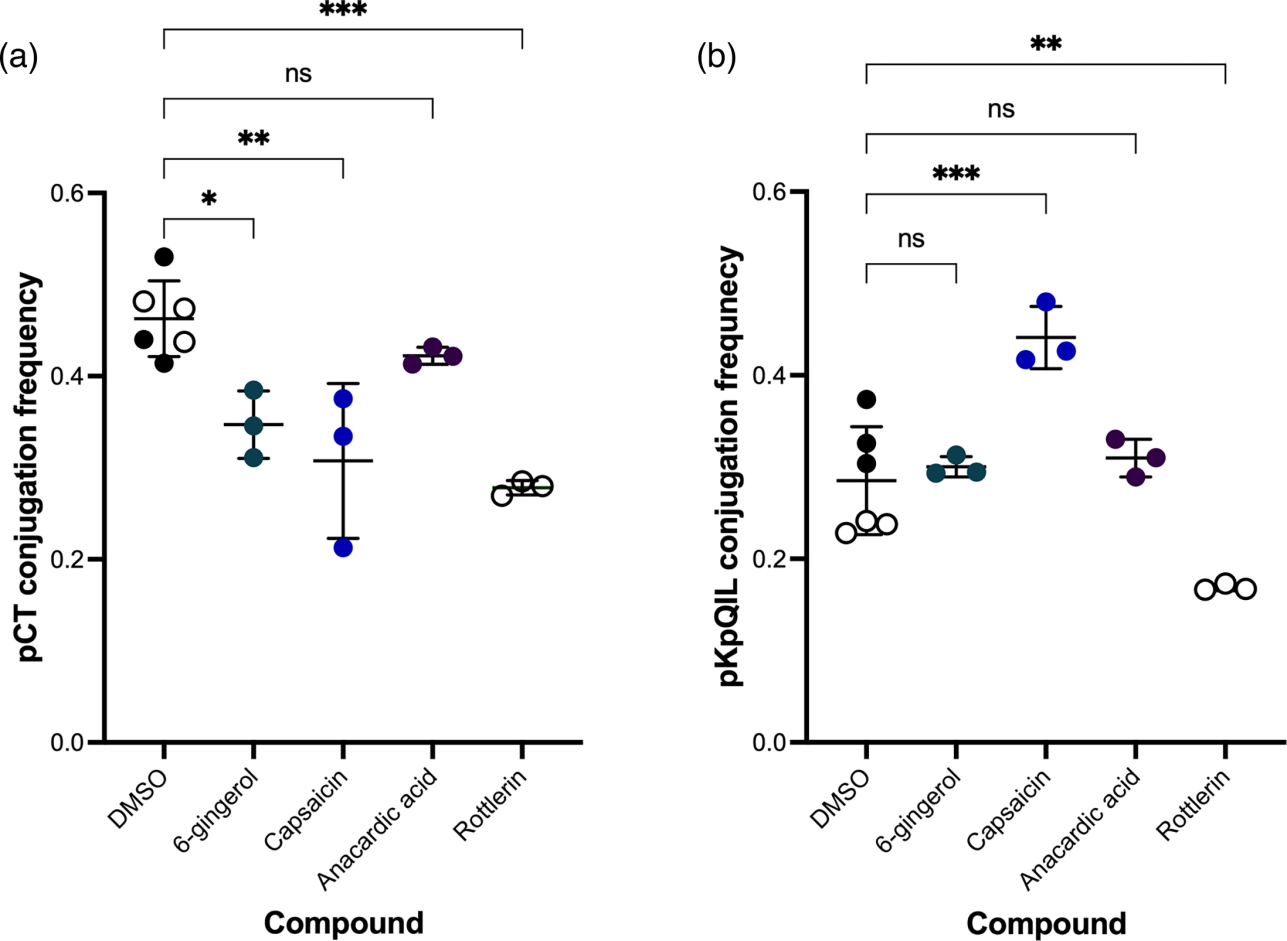
The effect of pure compounds on plasmid conjugation. (**a**) Conjugation frequency of pCT*gfp* from EC24 (*E. coli* EC958c carrying pCT*gfp*) to the recipient EC25 (*E. coli* EC958c mCherry). (**b**) Conjugation frequency of pKpQIL*gfp* from KP19 (*K. pneumoniae* Ecl8 carrying pKpQIL*gfp*) to the recipient KP18 (*K. pneumoniae* Ecl8 mCherry). The donor and recipient strains were mixed at a 1 : 1 ratio and then added to LB broth supplemented with 100 µg ml^−1^ of pure compounds or an equal volume of DMSO as vehicle control. Rottlerin was tested in separate experiments and, therefore, was compared to its DMSO control (indicated by clear circles). Conjugation assays were carried out for 24 h (*E. coli)* and 6 h (*K. pneumoniae)* at 37 °C and then analysed on the flow cytometer. A total of 10 000 bacterial events were recorded per sample. The conjugation frequencies were calculated as the number of dual fluorescent bacteria (transconjugants) divided by the mCherry-positive-only bacteria (recipients). Data presented are mean±standard deviation of three independent experiments, each consisting of four biological replicates. The conjugation frequencies of the plasmids treated with DMSO were compared to those treated with the pure compounds using one-way ANOVA with Dunnett’s test to correct for multiple comparisons. Significantly different results are indicated with * (*P*≤0.05) ** (*P*≤0.01) or *** (*P*≤0.001). ns, not significant.

Based on the literature, some pure compounds with known bioactive effects found in the food product extracts, or compounds with anticipated activity, were tested using the high-throughput conjugation assay (Fig. 2). All compounds were tested at 100 µg ml^−1^. As with the whole extracts, the conjugation of pCT in *E. coli* was more susceptible to inhibition than pKpQIL conjugation in *K. pneumoniae*. In *E. coli*, 6-gingerol (*P*=0.01), capsaicin (*P*=0.001), and rottlerin (*P*<0.001) significantly reduced the conjugation frequency of pCT compared to the DMSO control (Fig. 2a). In *K. pneumoniae*, only rottlerin (*P*=0.004) significantly reduced the conjugation frequency of pKpQIL, whilst 6-gingerol had no significant impact (*P*=0.962) and capsaicin significantly increased pKpQIL conjugation (*P*<0.001) (Fig. 2b). At 100 µg ml^−1^, anacardic acid had no significant effect on either pCT and pKpQIL conjugation frequencies (*P=*0.57 and *P*=0.823). At 100 µg ml^−1^, none of the pure compounds significantly affected the fluorescence of *E. coli* and *K. pneumoniae* cells compared to DMSO control (Fig. S2 and Table S2).

**Fig. 3.**
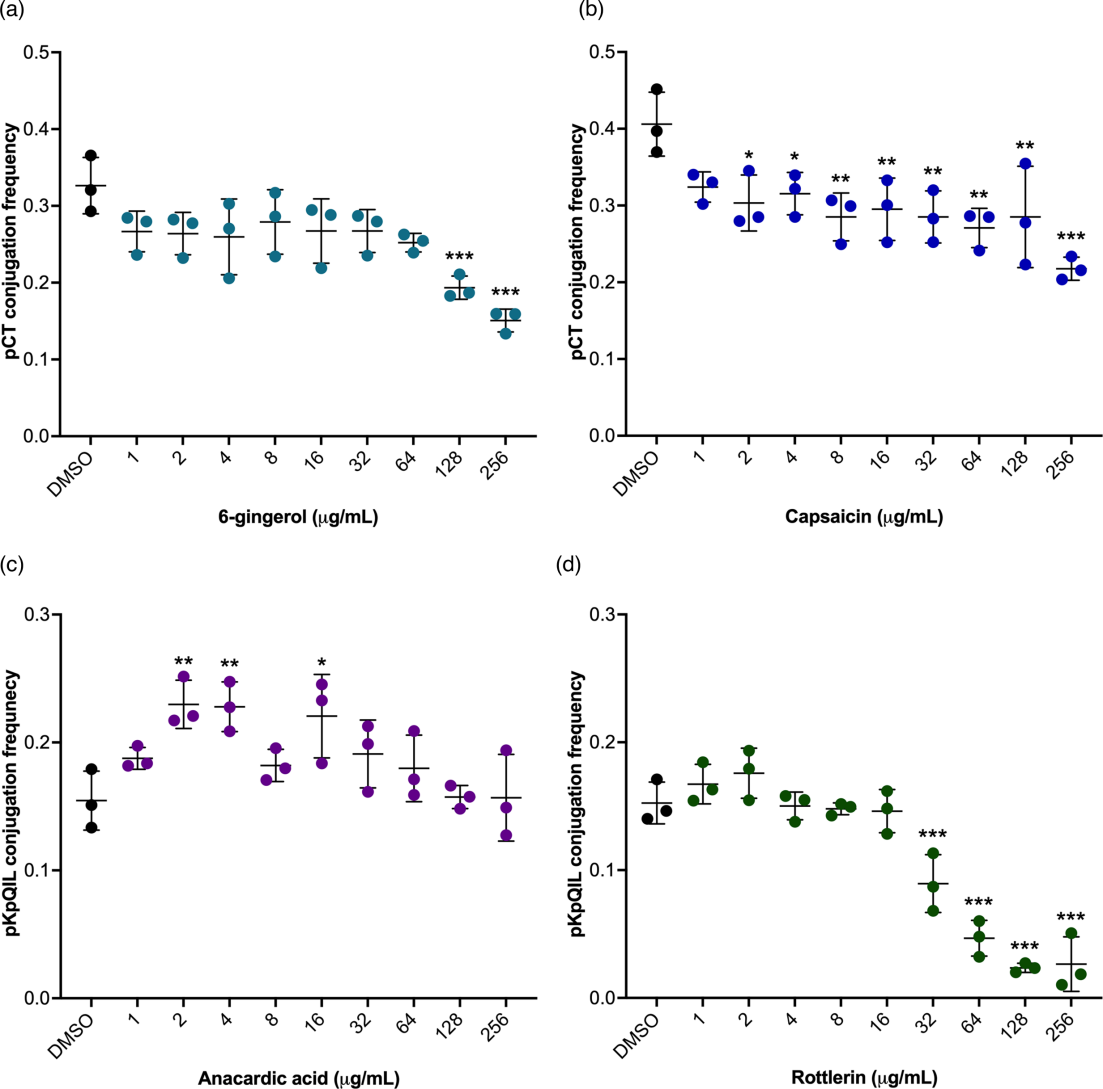
Dose–response of pure compounds on plasmid conjugation in *Escherichia coli* and *Klebsiella pneumoniae* measured by flow cytometry. The conjugation frequency of pCT*gfp* from EC24 (*E. coli* EC958c carrying pCT*gfp*) to the recipient EC25 (*E. coli* EC958c mCherry) treated with (a) capsaicin and (b) 6-gingerol from 1 to 256 µg ml^−1^. The conjugation frequency of pKpQIL*gfp* from KP19 (*K. pneumoniae* Ecl8 carrying pKpQIL*gfp*) to the recipient KP18 (*K. pneumoniae* Ecl8 mCherry) treated with (c) anacardic acid and (d) rottlerin from 1 to 256 µg ml^−1^. The donor and recipient strains were mixed at a 1 : 1 ratio and then added to LB broth supplemented with pure compounds or an equal volume of DMSO as vehicle control. Conjugation assays were carried out for 24 h (*E. coli*) and 6 h (*K. pneumoniae*) at 37 °C and then analysed on the flow cytometer. A total of 10 000 bacterial events were recorded per sample. The conjugation frequencies were calculated as the number of dual fluorescent bacteria (transconjugants) divided by the mCherry-positive-only bacteria (recipients). Data presented are mean±standard deviation of three independent experiments, each consisting of four biological replicates. The conjugation frequencies of the plasmids treated with DMSO were compared to those treated with the pure compounds using one-way ANOVA with Dunnett’s test to correct for multiple comparisons. Only significantly different results are presented and are indicated with * (*P*≤0.05) ** (*P*≤0.01) or *** (*P*≤0.001).

To look more at the impacts of some compounds with the greatest activity in *E. coli* EC958c carrying pCT, dose–response curves were performed with 1–256 µg ml^−1^ of 6-gingerol and capsaicin concentrations. 6-gingerol significantly reduced pCT conjugation frequency at 128 and 256 µg ml^−1^ (Fig. 3a; *P*<0.001). However, at these concentrations, 6-gingerol also significantly reduced the overall number of fluorescent cells recorded in the bacterial population compared to the DMSO control (Fig. S3 and Table S3). Therefore, the reduction in the conjugation frequency of pCT treated with higher concentrations of capsaicin could be due to interference with the expression of GFP and mCherry proteins. Capsaicin produced a dose-dependent reduction in the conjugation frequency of pCT at 2 µg ml^−1^ and above (Fig. 3b). At concentrations of 64 µg ml^−1^ and above, capsaicin also significantly reduced the overall number of fluorescent cells detected within the bacteria population compared to the DMSO control (Fig. S3). Therefore, capsaicin concentrations of 2–32 µg ml^−1^ were effective in reducing pCT conjugation without affecting the fluorescence of the donor and recipient cells.

For *K. pneumoniae* Ecl8 carrying pKpQIL, two compounds were selected, rottlerin because it caused a significant decrease in pKpQIL conjugation frequency, and anacardic acid because it had no effect at 100 µg ml^−1^ and therefore was tested at a wider concentration range to look for an effect. At lower concentrations (2, 4, and 16 µg ml^−1^), anacardic acid significantly increased the conjugation frequency of pKpQIL in *K. pneumoniae* Ecl8 (Fig. 3c). However, at higher concentrations, anacardic acid had no significant impact on pKpQIL conjugation frequency (Fig. 3c). Additionally, none of the anacardic acid concentrations tested had a significant effect on the overall number of fluorescent cells compared to DMSO control (Fig. S3c). Rottlerin significantly reduced the conjugation frequency of pKpQIL in *K. pneumoniae* Ecl8 at 32 µg ml^−1^ and above compared to the DMSO control (Fig. 3d). The greatest reduction was seen upon treatment with 128 or 256 µg ml^−1^ of rottlerin. However, at 128 and 256 µg ml^−1^, rottlerin significantly reduced the number of fluorescent bacterial cells compared to the DMSO control (Fig. S3d). Nonetheless, 32 and 64 µg ml^−1^ rottlerin caused a significant decrease in pKpQIL conjugation frequency without affecting the number of fluorescent *K. pneumoniae* Ecl8 cells (Fig. S3d).

**Fig. 4.**
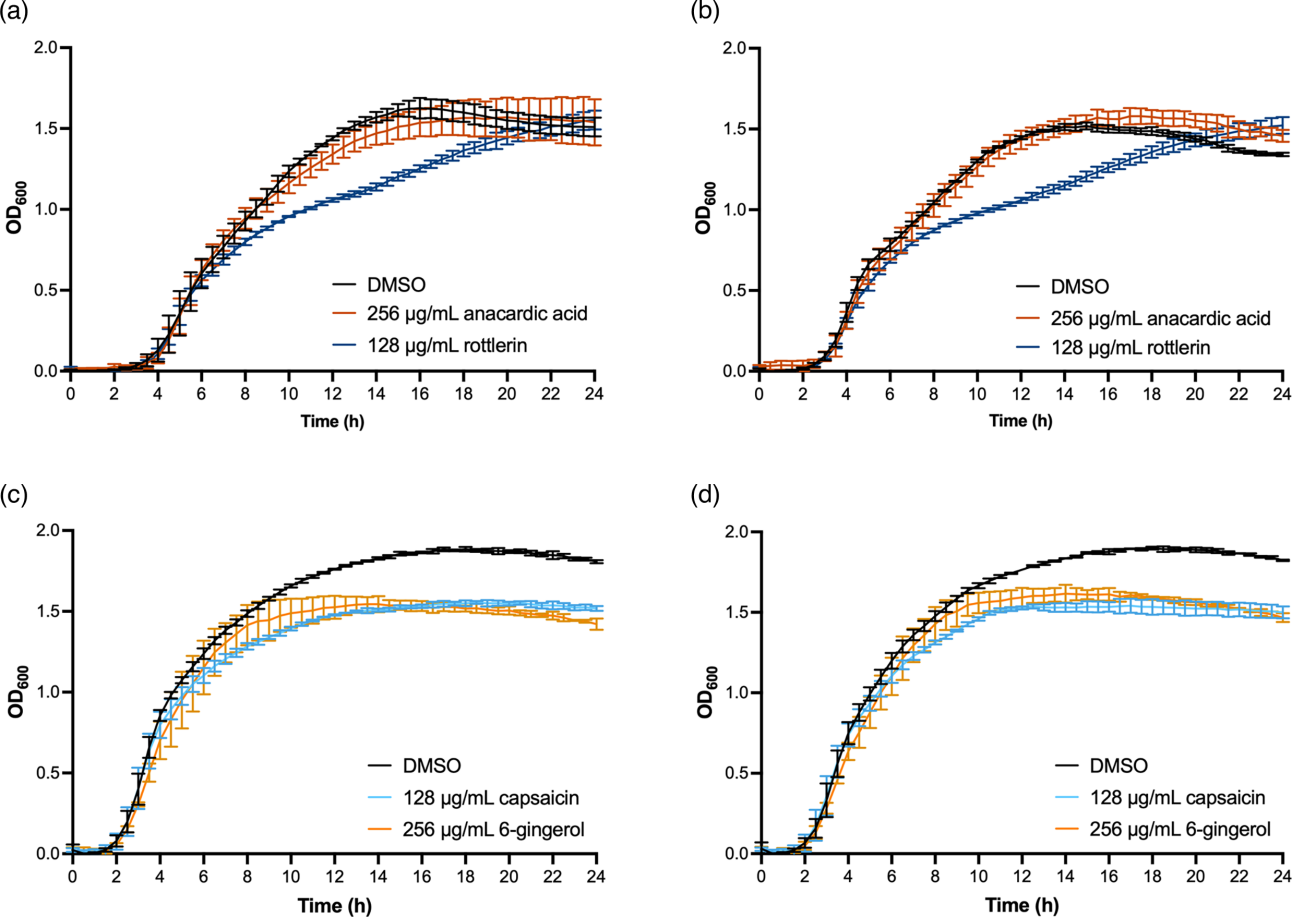
Growth kinetics of *Escherichia coli* and *Klebsiella pneumoniae* strains in the presence of natural product compounds. The growth of (a) KP18 (*K. pneumoniae* Ecl8 mCherry) and (b) KP19 (*K. pneumoniae* Ecl8 with pKpQIL*gfp*) over 24 h in LB broth supplemented with 256 µg ml^−1^ anacardic acid or 128 µg ml^−1^ rottlerin. The growth of (c) EC24 (*E. coli* EC958c with pCT*gfp*) and (d) EC25 (*E. coli* EC958c mCherry) over 24 h in LB broth supplemented with 128 µg ml^−1^ capsaicin or 256 µg ml^−1^ 6-gingerol. DMSO was added at the same volume as the highest compound concentration as vehicle control for all strains. The optical density at 600 nm (OD_600_) was measured every 30 min over 24 h at 37 °C with shaking (200 r.p.m.) in a plate reader. Data presented are mean±standard deviation of three independent experiments, each consisting of three biological replicates.

It is possible that potential anti-plasmid compounds could be inhibiting the growth of the donor or recipient strains, which would alter population density and both/either the number of cells able to donate or receive the plasmid. Antimicrobial susceptibility testing showed that none of the compounds inhibited the growth of the *E. coli* and *K. pneumoniae* strains (>512 µg ml^−1^) at concentrations above those tested in the conjugation assays (Table 2). To ensure that the pure compounds were not affecting the growth of the strains over the 6 and 24 h incubation used in the flow cytometry-based conjugation assays, *E. coli* and *K. pneumoniae* strains were grown in LB broth supplemented with the highest concentration of the pure compounds tested in the dose–response experiments. For KP18 and KP19, 256 µg ml^−1^ anacardic acid and 128 µg ml^−1^ rottlerin were tested because 256 µg ml^−1^ rottlerin adversely affected bacterial growth (Fig. 4). At 256 µg ml^−1^, anacardic acid had no impact on the growth of KP18 or KP19 over 24 h compared to the DMSO control (Fig. 4a, b). At 128 µg ml^−1^, rottlerin did not affect the growth of KP18 and KP19 during the medium log phase, up to ~6 h, which is the duration of the conjugation assay. However, it delayed the transition of both strains from the late log phase to the stationary phase, although both strains still reached the same final OD_600_ value as the DMSO control at the end of 24 h (Fig. 4a, b). For EC24 and EC25, 256 µg ml^−1^ 6-gingerol and 128 µg ml^−1^ capsaicin were tested because 256 µg ml^−1^ capsaicin reduced the growth of both strains. Neither 128 µg ml^−1^ capsaicin nor 256 µg ml^−1^ 6-gingerol affected bacterial growth during the log phase, but neither EC24 nor EC25 reached the same final density (Fig. 4c, d). Therefore, the reduction in pCT conjugation frequency in *E. coli* EC958c treated with high concentrations of capsaicin and 6-gingerol could be confounded by the high concentration’s impact on growth and cell density.

**Table 2.** Susceptibility of bacterial strains to natural product compounds

	MIC (μg ml^−1^)			
Strain	6-gingerol	Capsaicin	Anacardic acid	Rottlerin
Wild-type *E. coli* EC958c	>512	>512	nd	nd
*E. coli* EC958c *mCherry*	>512	>512	nd	nd
*E. coli* EC958c with pCT*gfp*	>512	>512	nd	nd
Wild-type *K. pneumoniae* Ecl8	nd	>512	>512	>512
*K. pneumoniae* Ecl8 *mCherry*	nd	>512	>512	>512
*K. pneumoniae* Ecl8 with pKpQIL*gfp*	nd	>512	>512	>512
*K. pneumoniae* with pCPE16_3 (clinical isolate)	>512	>512	>512	>512
*K. pneumoniae* ATCC 43816R (hygromycin-resistant recipient)	>512	>512	>512	>512

All strains were tested as three biological replicates. nd, not determined.

### Compounds impact on plasmid conjugation in a carbapenem-resistant *K. pneumoniae* clinical isolate

Next, the effect of the four compounds (capsaicin, anacardic acid, 6-gingerol, and rottlerin) was tested on KP10 (a clinical urine isolate of *K. pneumoniae*), which we have shown carries and readily transmits a 120 kb IncF plasmid with a *bla*_NDM-1_ carbapenem resistance gene, termed pCPE16_3 [36] The recipient strain was KP20, a previously generated hygromycin-resistant *K. pneumoniae* ATCC 43816R strain [36]. A 1 : 10 donor-to-recipient ratio of KP10 and KP20 was used for the conjugation assays. Each compound was tested at 100 µg ml^−1^, with a 1 h co-incubation of donor and recipient strains. Three parameters were explored: the number of transconjugants produced at the end of the 1 h incubation, the donor-to-recipient ratio after 1 h, and the conjugation frequency calculated as the number of transconjugants generated per recipient after 1 h.

**Fig. 5.**
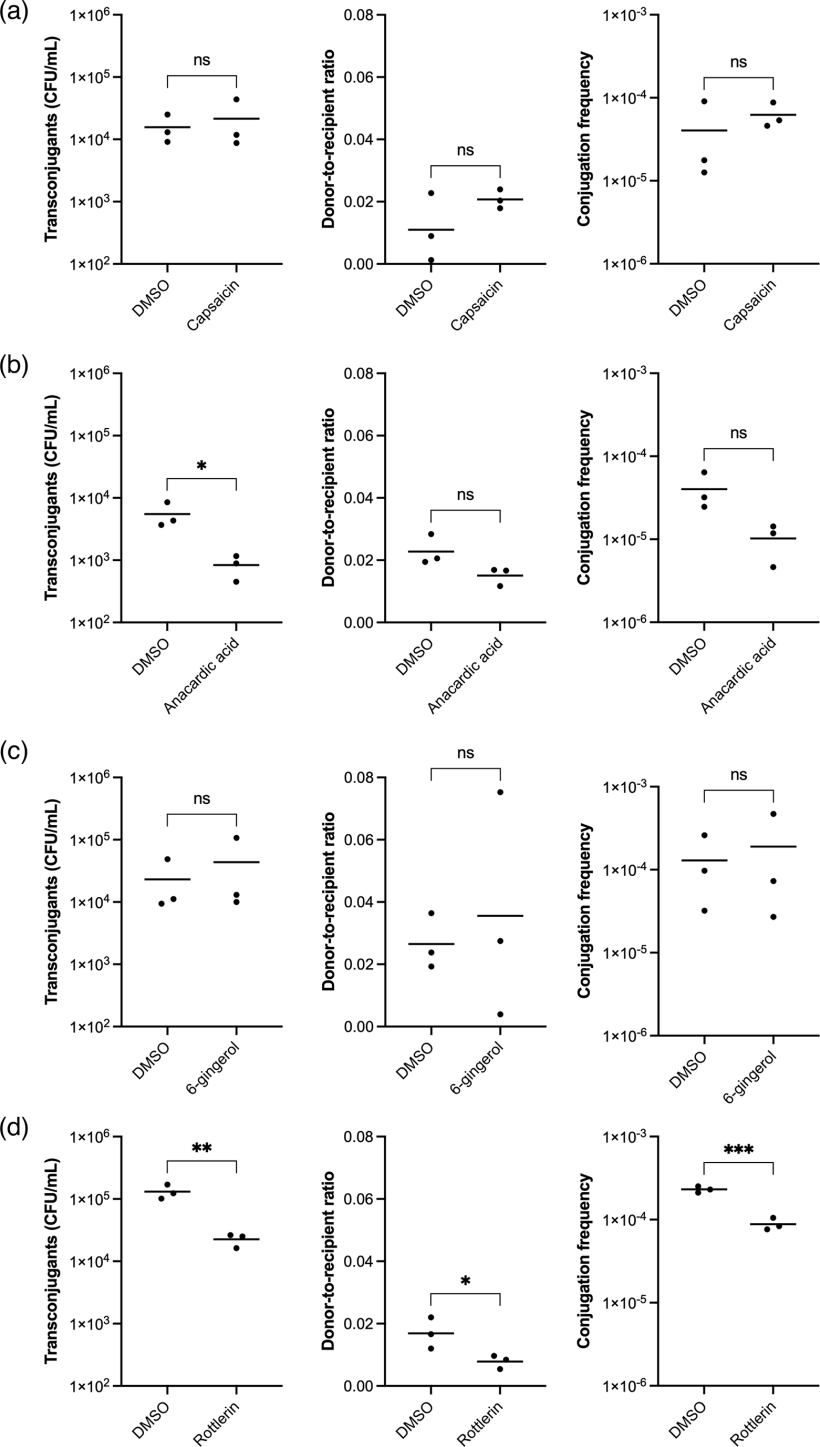
Liquid mating of KP10 (clinical *K. pneumoniae* isolate carrying the pCPE16_3 plasmid) and KP20 (hygromycin-resistant *K. pneumoniae* ATCC 43816 recipient strain) in the presence of pure compounds. The donor KP10 and the recipient KP20 strains were mixed at a 1 : 10 ratio and incubated in LB broth supplemented with 100 µg ml^−1^ of either (a) capsaicin, (**b**) anacardic acid, (**c**) 6-gingerol, or (d) rottlerin for 1 h at 37 °C. The DMSO control consisted of LB broth supplemented with an equal volume of DMSO as the natural product compounds. For each compound and DMSO control, the number of transconjugants (c.f.u. ml^−1^), the donor-to-recipient ratio, and the conjugation frequency (transconjugants per recipient) were calculated after 1 h of incubation. Data shown are the mean from three independent experiments, each carried out with four biological replicates. The mean of each parameter treated with DMSO control was compared to the mean of each parameter treated with the pure compound using two-tailed unpaired *t*-tests. Significant differences are indicated with * (*P*≤0.05), ** (*P*≤0.01), or *** (*P*≤0.001). ns, not significant.

For capsaicin, there was no change in the number of transconjugant bacteria, the donor-to-recipient ratio, or the pCPE16_3 conjugation frequency (Fig. 5a; *P*=0.4713, 0.2513, and 0.4446, respectively). This is in contrast with what was seen with pKpQIL, where the conjugation frequency was significantly higher compared to the DMSO control (Fig. 2b). Anacardic acid significantly reduced the number of transconjugant bacteria (*P*=0.0375), but had no significant effect on the donor-to-recipient ratio or conjugation frequency (Fig. 5b; *P*=0.2302 and 0.1937, respectively). This was comparable to pKpQIL, where anacardic acid also did not affect conjugation frequency (Fig. 2b). Comparable to pKpQIL, 6-gingerol did not affect any of the parameters for pCPE16_3 conjugation (Fig. 5c; *P*=0.3988, 0.6255, 0.6850, respectively). Interestingly, rottlerin significantly reduced all three tested parameters. It significantly reduced the total number of transconjugant bacteria from 1.32×10^5^ in DMSO to 2.25×10^4^ (*P*=0.006), the donor-to-recipient ratio from 0.0169 to 0.00783 (*P*=0.045), and the conjugation frequency of pCPE16_3 from 2.313×10^−4^ in DMSO to 8.83×10^−5^ (Fig. 5d). Antimicrobial susceptibility testing showed that both KP10 and KP20 had an MIC of >512 µg ml^−1^ for rottlerin (Table 2). However, since rottlerin reduced the ratio of KP10 to KP20, and impacted on the growth kinetics of KP18 and KP19, the impact of 100 µg ml^−1^ rottlerin or an equal volume of DMSO on the viability of KP10 and KP20 cells was determined following 1 h incubation. Compared to DMSO control, KP10 formed fewer colonies after 1 h of incubation with 100 µg ml^−1^ rottlerin; however, the difference was not statistically significant. This suggested that sub-MIC rottlerin may have affected KP10 growth during the conjugation time frame (Fig. S4).

## Discussion

The growing threat of AMR necessitates the search for alternative strategies to combat the spread and prevalence of AMR genes. Anti-plasmid compounds that interfere with plasmid conjugation or stability are being explored as a potential way to address AMR. Since natural products possess a wide range of biological activities, they offer a promising source of anti-plasmid compounds. To that end, we investigated the effect of natural product extracts and some of their reported bioactive constituents on plasmid conjugation.

We found that most natural product extracts reduced plasmid conjugation in both *E. coli* and *K. pneumoniae*. To identify the possible compounds responsible for reducing plasmid conjugation within the natural product extracts, we tested the major and widely reported bioactive compounds present in some of these extracts. We found that capsaicin and 6-gingerol significantly reduced pCT transmission in *E. coli* EC958c. Previous work showed that 6-gingerol reduced the transfer of the IncN pKM101, IncP pUB307, and IncI2 TP114 plasmids, while capsaicin reduced the transfer of the IncI2 TP114, IncW R7K, IncP pUB307 and IncN pKM101 plasmids in *E. coli* K12 J53, without antibacterial effects on Gram-negative bacteria [37]. Based on plasmid replicon typing, the IncI2 plasmid TP114 and the IncK plasmid pCT belong to the I complex [38,39]. Therefore, the impact of 6-gingerol and capsaicin on pCT conjugation is comparable to the impact on TP114 conjugation. In a different study, rottlerin reduced the conjugation of the IncN pKM101, IncI2 TP114, IncP pUB307, and IncX2 R6K plasmids in *E. coli* K-12 J53 [40]. In agreement, our data showed that in a clinical *E. coli* EC958c isolate with a veterinary plasmid [17], rottlerin also reduced the conjugation of the IncK plasmid pCT, which belongs to the same I complex as TP114. The effect of anacardic acid on plasmid conjugation has not been reported before, and we found that it did not significantly affect plasmid conjugation. However, at low concentrations, it increased pKpQIL conjugation in *K. pneumoniae* Ecl8. Some anti-plasmid compounds have plasmid-specific effects [26,41], therefore, anacardic acid may have activity (increase or decrease conjugation) in other plasmid types.

This study used a fluorescent reporter assay to monitor plasmid conjugation by flow cytometry. Using fluorescent reporters to monitor plasmid conjugation increases throughput; however, consideration must be given to the potential impact of compounds on the expression and function of fluorescent proteins. For example, during conjugation, black pepper, ginger, turmeric, and kamala extracts significantly increased the number of non-fluorescent *E. coli* cells, whereas *K. pneumoniae* cells were less prone to reduction in fluorescence. Therefore, the apparent decrease in pCT conjugation frequency in *E. coli* treated with the natural product extracts could partly be due to the significant reduction of fluorescent cells. At 100 µg ml^−1^, none of the pure compounds significantly affected the fluorescence of *E. coli* and *K. pneumoniae* cells. However, at higher concentrations (128 and 256 µg ml^−1^), capsaicin, 6-gingerol, and rottlerin significantly reduced the fluorescence of *E. coli* and *K. pneumoniae* cells. Certain natural product compounds display intrinsic fluorescence or quenching [42], hence, the apparent reduction in conjugation frequency by higher concentrations of natural product compounds is likely due to fluorescence quenching.

The activity of anti-plasmid compounds could also be influenced by plasmid–host combination. Some bacterial host strains can acquire and maintain certain plasmids without a fitness cost [43]. These successful host–plasmid pairings contribute significantly to the global spread of AMR genes [44]. Despite their prevalence, there is still limited knowledge of what factors influence plasmid transfer in these successful plasmid–host combinations [45]. Therefore, understanding the intricacies of the plasmid–host relationship is important in developing effective strategies to combat plasmid-mediated antibiotic resistance. The ideal anti-plasmid compound would have broad-range activity against different host strains and plasmids; however, owing to the diversity of plasmids and their relationship with the host, this may prove difficult. Nonetheless, identifying compounds that target globally disseminating plasmid–host combinations could be an attractive strategy to prevent the spread of AMR plasmids.

Overall, we found that the potency of the natural product compounds was low as the density and number of transconjugant cells were still too high following treatment with the compounds. Therefore, the natural product compounds investigated in this study are unlikely to curb the spread of AMR plasmids in bacterial populations. Nonetheless, the data in this study suggests that certain natural product compounds like rottlerin could provide a chemical scaffold for further developing novel anti-plasmid compounds using structure–activity relationship studies.

## supplementary material

10.1099/mic.0.001496 Supplementary Material 1.

## Abbreviations

AMR: antimicrobial resistance
DMSO: dimethylsulfoxide
ESBL: extended spectrum beta-lactamase
GFP: green fluorescent protein
KPC: *Klebsiella pneumoniae* carbapenemase
LB: Luria-Bertani
MIC: minimum inhibitory concentration
OD: optical density
