# Supplementary material for: Natural products from food sources can alter the spread of antimicrobial resistance plasmids in Enterobacterales
Source: Microbiology (Reading). 2024 Aug 27;170(8):001496. doi: 10.1099/mic.0.001496 (PMC11541548; doi:10.1099/mic.0.001496)
Supplement: Supplementary Material 1. [file mic-170-01496-s001.pdf]

Supplementary data

Natural products from food sources can alter the spread of antimicrobial resistance plasmids in *Enterobacterales*

Ilyas Alav[1], Parisa Pordelkhaki[1], Judith Rodriguez-Navarro[2], Onalenna Neo[3], Celia Kessler[1], Ruth Jesujobalayemi Awodipe[4], Poppy Cliffe[1], Nivethanaa Pulavan,[1] Huba L. Marton[1], Simon Gibbons[5], Michelle M.C. Buckner[1#]

[#] Corresponding Author

[1] Institute of Microbiology and Infection, College of Medical and Dental Sciences, University of Birmingham.

[2] Department of Microbiology, Hospital de la Santa Creu i Sant Pau, Institut d'Investigació Biomèdica Sant Pau (IIB Sant Pau), Sant Quintıí 89, E-08041 Barcelona, Spain

[3] School of Dentistry, College of Medical and Dental Sciences, University of Birmingham. (Current address: Wellcome Sanger Institute, Wellcome Trust Genome Campus, Hinxton, Saffron Walden CB10 1RQ)

[4] Warwick Medical School, University of Warwick

[5] Natural & Medical Sciences Research Center, University of Nizwa, P.O. Box 33, Birkat Al Mauz, Nizwa 616, Sultanate of Oman

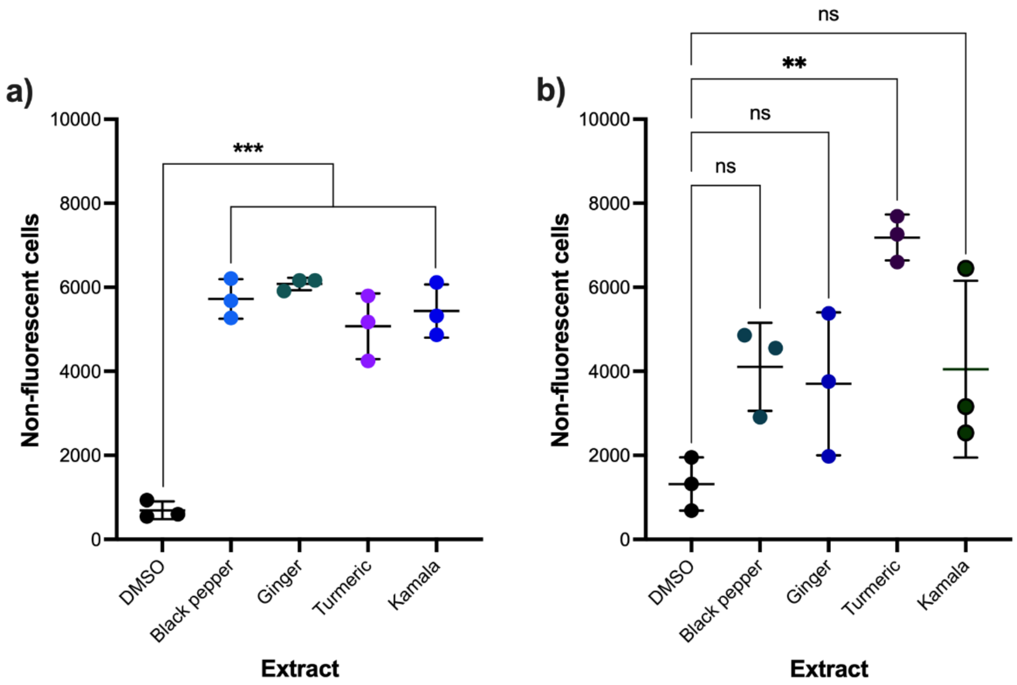

Figure S1. The effect of extracts on the fluorescence of GFP and mCherry-expressing *Escherichia coli* and *Klebsiella pneumoniae* cells. a) The number of non-fluorescent donor EC24 (*E. coli* EC958c with pCT*gfp*) and recipient EC25 (*E. coli* EC958c mCherry) cells and **b)** the number of non-fluorescent recipient KP18 (*K. pneumoniae* Ecl8 mCherry) and donor KP19 (*K. pneumoniae* Ecl8 with pKpQIL*gfp*) cells. The donor and recipient strains were mixed at a 1:1 ratio. The mixed cells were added to LB broth supplemented with 0.25 mg/mL of extracts or an equal volume of DMSO as vehicle control before being incubated for 24 hours at 37 °C. A total of 10,000 bacterial events were recorded per sample. Non-fluorescent cells were those lacking GFP or mCherry expression (GFP-/mCherry-). Data presented are the mean ± standard deviation of three independent experiments, each consisting of four biological replicates. The mean number of non-fluorescent cells incubated with DMSO were compared to those treated with the extracts using one-way ANOVA with Dunnett's test to correct for multiple comparisons. Significantly different results are indicated with * ($P \leq 0.05$) or *** ($P \leq 0.001$). ns, not significant

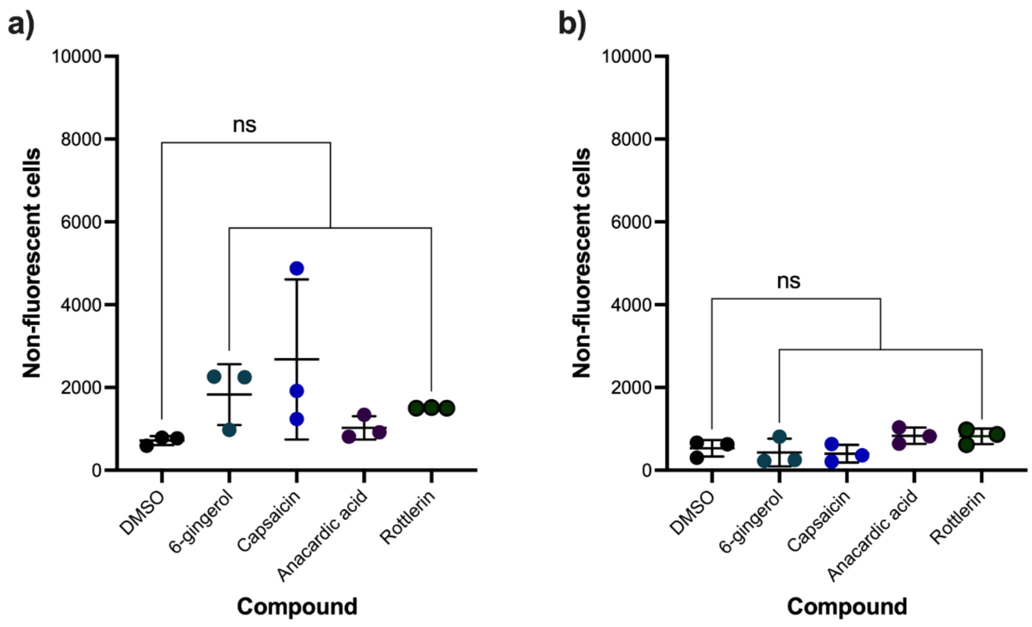

Figure S2. The effect of pure compounds on the fluorescence of GFP and mCherry-expressing *Escherichia coli* and *Klebsiella pneumoniae* cells. a) The number of non-fluorescent donor EC24 (*E. coli* EC958c with pCT*gfp*) and recipient EC25 (*E. coli* EC958c mCherry) cells and **b)** the number of non-fluorescent recipient KP18 (*K. pneumoniae* Ecl8 mCherry) and donor KP19 (*K. pneumoniae* Ecl8 with pKpQIL*gfp*) cells. The donor and recipient strains were mixed at a 1:1 ratio. The mixed cells were added to LB broth supplemented with 100 µg/mL of pure compound or an equal volume of DMSO as vehicle control before incubating for 24 hours (*E. coli)* and 6 hours (*K. pneumoniae)* at 37 °C. A total of 10,000 bacterial events were recorded per sample. Non-fluorescent cells were those lacking GFP or mCherry expression (GFP-/mCherry-). Data presented are the mean ± standard deviation of three independent experiments, each consisting of four biological replicates. The mean number of non-fluorescent cells incubated with DMSO was compared to those treated with the pure compounds using one-way ANOVA with Dunnett's test to correct for multiple comparisons. ns, not significant

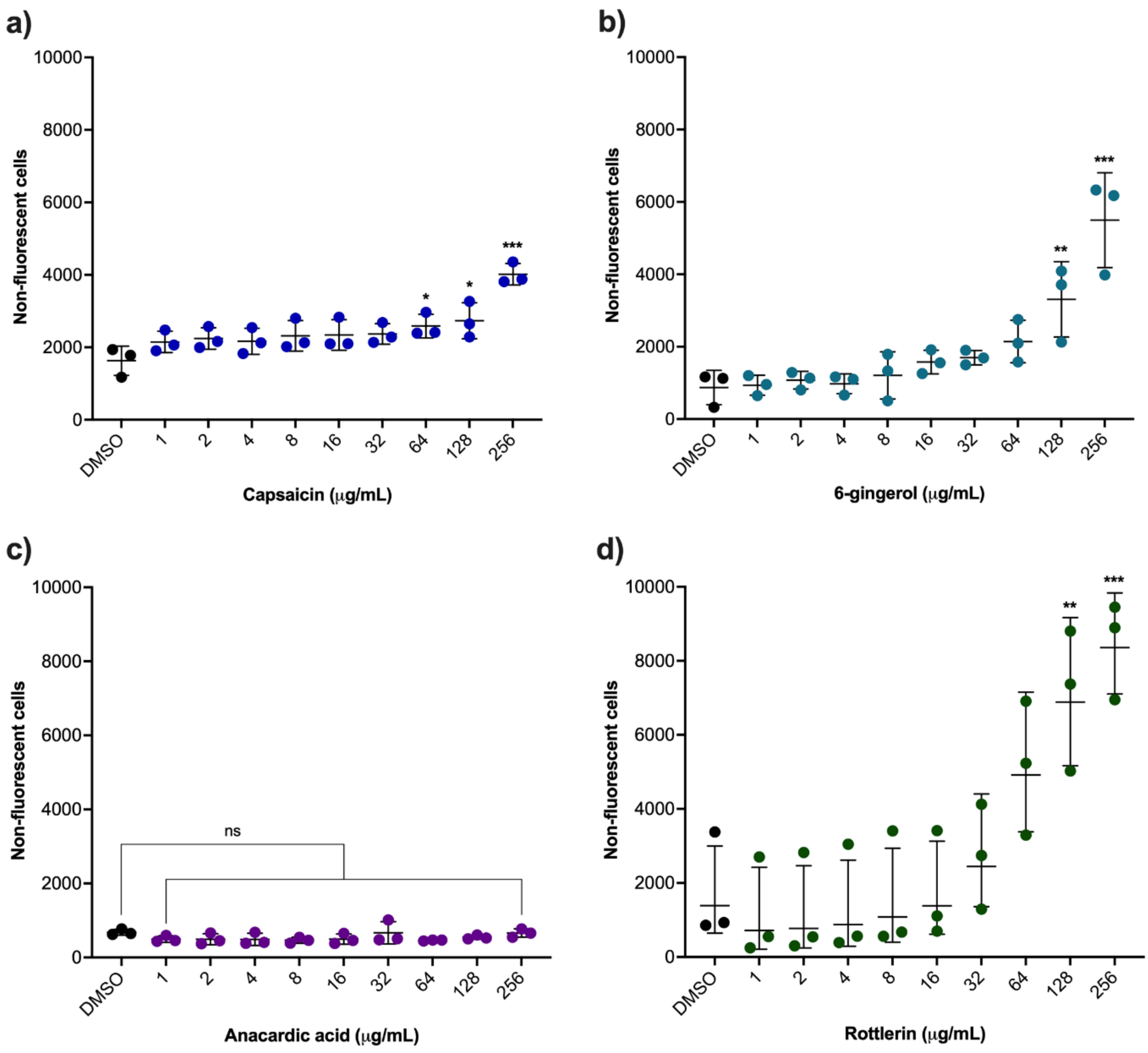

Figure S3. Dose-response of pure compounds on the fluorescence of GFP and mCherry-expressing *Escherichia coli* and *Klebsiella pneumoniae* cells. The number of non-fluorescent donor EC24 (*E. coli* EC958c with pCT*gfp*) and recipient EC25 (*E. coli* EC958c mCherry) treated with **a)** capsaicin and **b)** 6-gingerol. The number of non-fluorescent recipient KP18 (*K. pneumoniae* Ecl8 mCherry) and donor KP19 (*K. pneumoniae* Ecl8 with pKpQIL*gfp*) cells treated with **c)** anacardic acid and **d)** rottlerin. The donor and recipient strains were mixed at a 1:1 ratio. The mixed cells were added to LB broth supplemented with the indicated concentrations of the pure compound or an equal volume of DMSO as vehicle control before being incubated for 24 hours at 37 °C. A total of 10,000 bacterial events were recorded per sample. Non-

fluorescent cells were those lacking GFP or mCherry expression (GFP-/mCherry-). Data presented are the mean ± standard deviation of three independent experiments, each consisting of four biological replicates. The mean number of non-fluorescent cells incubated with DMSO was compared to those treated with the pure compounds using one-way ANOVA with Dunnett's test to correct for multiple comparisons. Significantly different results are indicated with * ($P \leq 0.05$), ** ($P \leq 0.01$), or *** ($P \leq 0.001$). ns, not significant

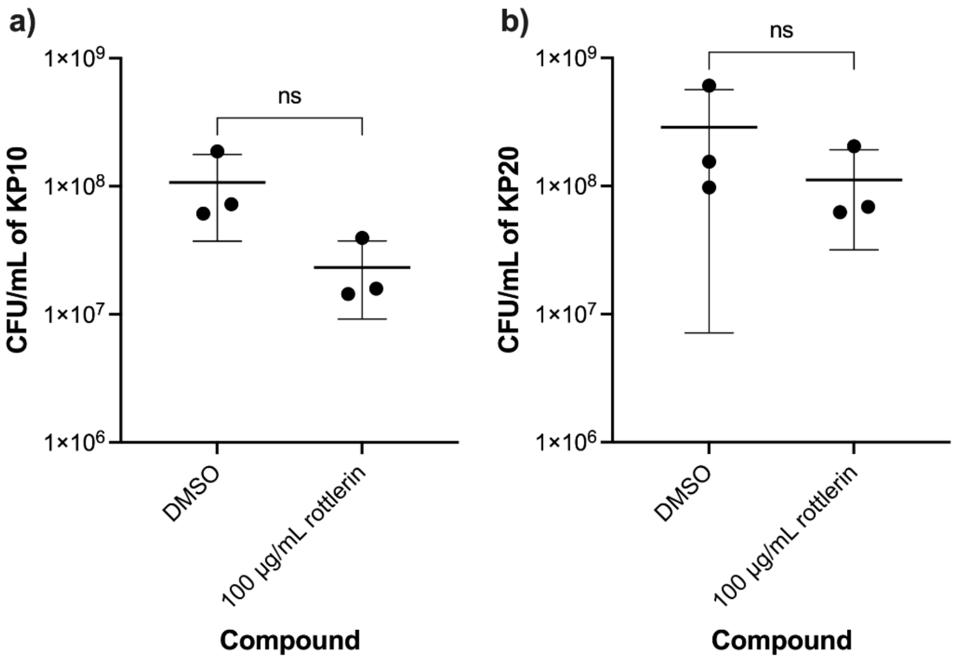

Figure S4. The effect of rottlerin on the viability of clinical *Klebsiella pneumoniae* strains. The CFU/mL of **a)** KP10 (clinical carbapenem-resistant *K. pneumoniae* strain carrying the IncF pCPE16_3 plasmid) and **b)** KP20 (hygromycin-resistant *K. pneumoniae* ATCC 43816R recipient strain), following incubation in LB broth supplemented with 100 µg/mL rottlerin or an equal volume of DMSO as vehicle control for one-hour at 37 °C. Data presented are the mean ± standard deviation of three independent experiments, each consisting of four biological replicates. The mean CFU/mL of the strains following treatment with 100 µg/mL rottlerin was compared to DMSO control using two-tailed unpaired t-tests. ns, not significant

Supplementary Table S1. The impact of the natural product extracts on the fluorescence of *Escherichia coli* and *Klebsiella pneumoniae* cells. The data shown are the mean values of three independent experiments, each consisting of four biological replicates. Dual fluorescent cells expressed mCherry and GFP, whereas non-fluorescent cells lacked mCherry and GFP expression.

a) The number of dual fluorescent and non-fluorescent *E. coli* cells after 24-hour incubation in LB broth supplemented with 0.25 mg/mL natural extract or an equal volume of DMSO. A total of 10,000 bacterial events were recorded per sample.

**Extract**	**Dual fluorescent cells**			**Non-fluorescent cells**		
DMSO	1201.75	1264.75	1906	599.5	933.75	545.75
Black pepper	265.25	202.75	184	5274	5681.25	6210.25
Ginger	295.75	358.75	295.33	6166.25	5911	6166
Turmeric	253.25	408.5	582	5796.75	5173.5	4247
Kamala	363.75	262.25	840.25	6116.75	5320.25	4867.25

b) The number of dual fluorescent and non-fluorescent *K. pneumoniae* cells after 6-hour incubation in LB broth supplemented with 0.25 mg/mL natural extract or an equal volume of DMSO. A total of 10,000 bacterial events were recorded per sample.

**Extract**	**Dual fluorescent cells**			**Non-fluorescent cells**		
DMSO	938.5	676.25	1131	1320.5	1953.5	684.75
Black pepper	284	535.25	190	4859	2907.25	4555.85
Ginger	926.5	566.25	1719.5	3755.75	5377	1977
Turmeric	129.25	250.25	189.25	7688.5	6603	7262.5
Kamala	280.75	374.25	265.5	6452.25	2535.5	3163.25

Supplementary Table S2. The impact of the pure compounds on the fluorescence of *Escherichia coli* and *Klebsiella pneumoniae* cells. The data shown are the mean values of three independent experiments, each consisting of four biological replicates. Dual fluorescent cells expressed mCherry and GFP, whereas non-fluorescent cells lacked mCherry and GFP expression.

a) The number of dual fluorescent and non-fluorescent *E. coli* cells after 24-hour incubation in LB broth supplemented with 100 µg/mL pure compound or an equal volume of DMSO. A total of 10,000 bacterial events were recorded per sample.

Compound	Dual fluorescent cells			Non-fluorescent cells		
DMSO	1288.5	1371.5	1184.75	787	776	594
6-gingerol	659.5	682.5	1198.75	2262.5	2246.75	980
Capsaicin	666.25	238	1056.25	1920	4876.5	1242
Anacardic acid	1273.5	1453.25	872	813.75	919.25	1343.5
Rottlerin	1159	1112.25	1167.75	1503.5	1516.75	1501.25

b) The number of dual fluorescent and non-fluorescent *K. pneumoniae* cells after 6-hour incubation in LB broth supplemented with 100 µg/mL pure compound or an equal volume of DMSO. A total of 10,000 bacterial events were recorded per sample.

Compound	Dual fluorescent cells			Non-fluorescent cells		
DMSO	1842.5	1833	1023.25	626.75	302	667.5
6-gingerol	1730.5	1914.67	1528.75	815	223.75	248.5
Capsaicin	2577	3146	2948.75	359.25	211.75	633
Anacardic acid	810.25	753	802	1035.5	645	823
Rottlerin	790.25	653.75	779.25	863.75	617.33	979.75

Supplementary Table S3. The impact of pure compounds at 1-256 µg/mL on the fluorescence of *Escherichia coli* and *Klebsiella pneumoniae* cells. The data shown are the mean values of three independent experiments, each consisting of four biological replicates. Dual fluorescent cells expressed mCherry and GFP, whereas non-fluorescent cells lacked mCherry and GFP expression. A total of 10,000 bacterial events were recorded per sample.

a) The number of dual fluorescent and non-fluorescent *E. coli* cells after 24-hour incubation in LB broth supplemented with 1-256 µg/mL capsaicin or an equal volume of DMSO. A total of 10,000 bacterial events were recorded per sample.

Capsaicin concentration (µg/mL)	Dual fluorescent cells			Non-fluorescent cells		
0	806	848.25	680.75	1174.25	1779.75	1937.25
1	686.25	704.75	628.25	1905.25	2063.25	2479.75
2	692.25	598.25	563.75	1994.25	2163.25	2573
4	714.25	685.75	578.75	1829.5	2126.25	2545.25
8	562.75	685.5	494.75	2128	2020.75	2800.75
16	649.75	638	497.25	2100.25	2098.25	2828.75
32	628.25	602.75	542	2135.25	2284	2685.25
64	532	627.75	475	2411	2389.75	2966.5
128	711.5	573.5	446.25	2287.5	2645.5	3268
256	297.25	407	397.25	4356.5	3879	3814.5

b) The number of dual fluorescent and non-fluorescent *E. coli* cells after 24-hour incubation in LB broth supplemented with 1-256 µg/mL 6-gingerol or an equal volume of DMSO. A total of 10,000 bacterial events were recorded per sample.

6-gingerol concentration (µg/mL)	Dual fluorescent cells			Non-fluorescent cells		
0	353	209.25	200.75	879.25	1039	561.75
1	340.75	248	291.5	1105.5	1669.25	1072.75
2	407.5	193.25	288.5	879.25	1427.5	856
4	468.5	343.75	319.25	995.5	1615	1146
8	333.5	320	246.5	1339.5	2012.75	1217.25
16	378	145	231	1084	1936	982.5
32	334.5	171.75	161.25	1709.25	2476.75	1698.5
64	211.75	54.5	74.75	3348.25	5975.25	3006.75
128	60.75	9.25	30.25	5460	8108.75	6068.25
256	17.75	21.25	13	7729.5	8395.75	7958.25

c) The number of dual fluorescent and non-fluorescent *K. pneumoniae* cells after 6-hour incubation in LB broth supplemented with 1-256 µg/mL anacardic acid or an equal volume of DMSO. A total of 10,000 bacterial events were recorded per sample.

**Anacardic acid concentration (µg/mL)**	**Dual fluorescent cells**			**Non-fluorescent cells**		
0	325	394.75	461.75	771	648	623.75
1	512.75	395.75	561.75	596.25	437.75	455.5
2	440	466.75	743.5	662	451.5	371.5
4	525.25	587.5	565	679.25	407.75	384.25
8	611	401.75	462.75	546.25	465	386.5
16	425.25	492.25	670	650.75	459.75	378.75
32	492.5	488.25	444	1013.75	506.5	479
64	325	394.75	461.75	771	648	623.75
128	512.75	395.75	561.75	596.25	437.75	455.5
256	440	466.75	743.5	662	451.5	371.5

d) The number of dual fluorescent and non-fluorescent *K. pneumoniae* cells after 6-hour incubation in LB broth supplemented with 1-256 μg/mL rottlerin or an equal volume of DMSO. A total of 10,000 bacterial events were recorded per sample.

**Rottlerin concentration (μg/mL)**	**Dual fluorescent cells**			**Non-fluorescent cells**		
0	200.75	274	108.75	854.75	930.5	3375
1	262.75	269.75	75.5	553.5	246.75	2703
2	313.5	291	107.25	544.25	301.25	2823
4	289.75	247.5	91.75	564	389	3045
8	245.25	289.25	87	671.5	558.25	3407.5
16	224	315.25	99.25	1110.25	700.5	3414.25
32	189.5	202.5	37.75	2743.75	1293.25	4124.75
64	101.75	157.5	16	5234.5	3291.25	6909
128	45	77.5	3.25	7372.25	5025	8801.75
256	10	51.25	4	8892.25	6951	9449.25
